# Cerebellar atrophy on top of motor neuron compromise as indicator of late-onset GM2 gangliosidosis

**DOI:** 10.1007/s00415-021-10492-y

**Published:** 2021-03-09

**Authors:** Hans Thomas Hölzer, Felix Boschann, Julia B. Hennermann, Gabriele Hahn, Andreas Hermann, Maja von der Hagen, Victoria Tüngler

**Affiliations:** 1 Abteilung Neuropädiatrie, Medizinische Fakultät Carl Gustav Carus, Technische Universität Dresden, Dresden, Germany; 2 Institute of Medical Genetics and Human Genetics, Charité – Universitätsmedizin Berlin, corporate member of Freie Universität Berlin and Humboldt-Universität zu Berlin, Berlin, Germany; 3 Department of Pediatric and Adolescent Medicine, University Medical Centre Mainz, Mainz, Germany; 4 Bereich Kinderradiologie, Institut und Poliklinik für Diagnostische und Interventionelle Radiologie, Medizinische Fakultät Carl Gustav Carus, Technische Universität Dresden, Dresden, Germany; 5 Translational Neurodegeneration Section “Albrecht-Kossel”, Department of Neurology, University Medical Centre Rostock, University of Rostock, Rostock, Germany; 6 UniversitätsCentrum für Seltene Erkrankungen, Medizinische Fakultät Carl Gustav Carus, Technische Universität Dresden, Dresden, Germany

**Fig. 1:**
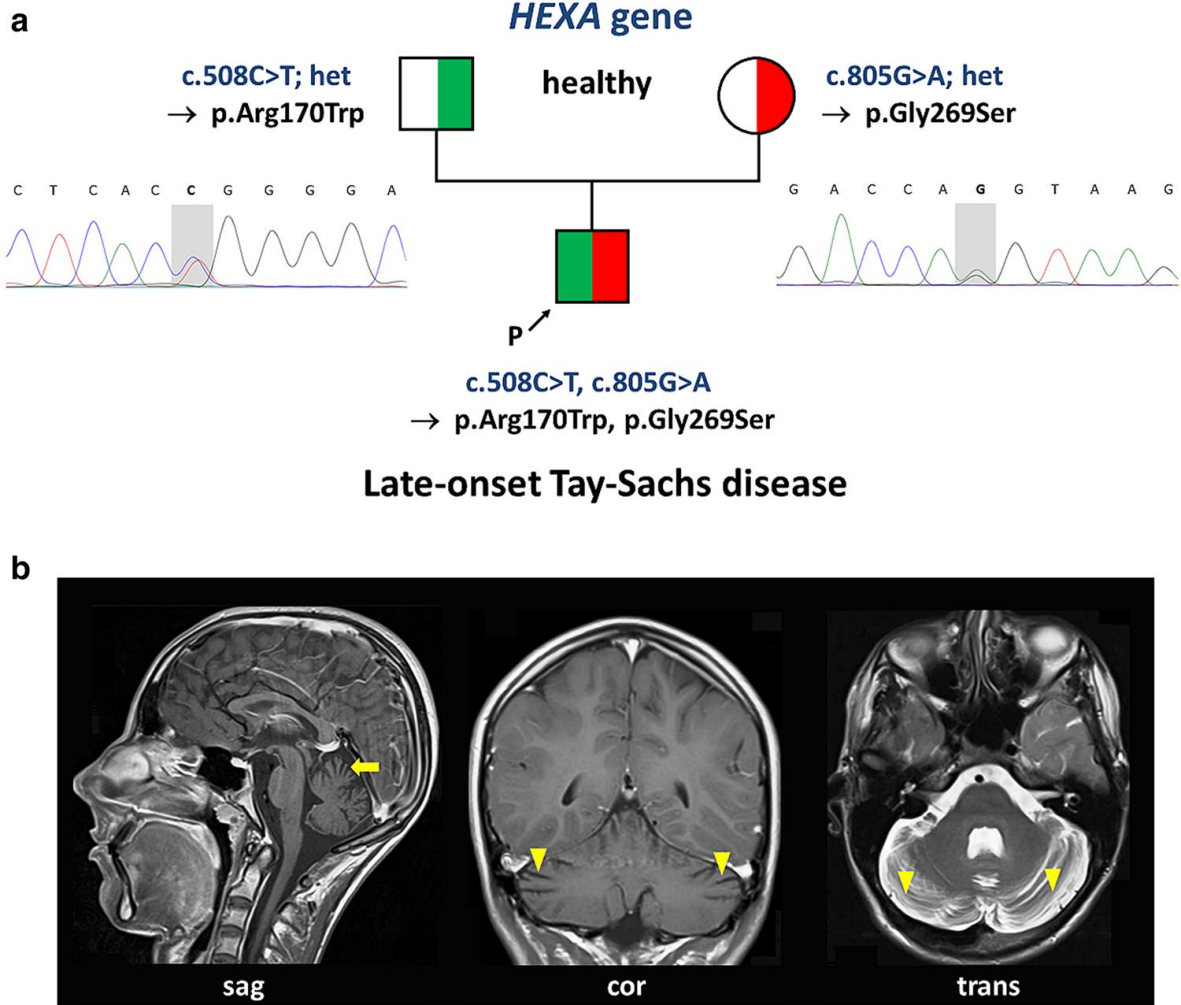
Genetic and clinical findings. **a** Pedigree and electropherograms showing the mutations in the *HEXA* gene. The affected patient inherited the two pathogenic variants c.508C>T (paternal) and c.805G>A (maternal). **b** Magnetic resonance brain images of the index patient at the age of 16 years in T1-weighted contrast-enhanced sagittal (sag) and coronal (cor) planes as well as T2-weighted transverse view (trans) show cerebellar atrophy with widening of the cerebellar cortical sulci (arrows) and interfolial spaces (arrow heads)

Dear Sirs,

An 18-year-old-male of healthy non-consanguineous parents presented with slowly progressive muscle weakness. The onset of his clinical symptoms began at the age of 13 years with fatigue and muscle pain. 1 year later he complained about increasing walking difficulties. On physical examination the patient exhibited cachexia, dysarthria, scoliosis, generalized muscular atrophy, bilateral symmetric hyperreflexia, a mild ataxic gait as well as subtle bilateral action tremor. Electromyography revealed signs of diffuse denervation reminiscent of lower motor neuron degeneration or anterior horn disease. Magnetic resonance imaging (MRI) of the brain at the age of 16 years showed moderate diffuse cerebellar atrophy without supratentorial cerebral atrophy (Fig. 1). Serum creatine kinase (CK) levels were repeatedly elevated (max. 2.5-fold).

The patient was included in TRANSLATE-NAMSE, an innovation project of the German Federal Joint Committee to improve the care of patients with rare diseases. The study was conducted with approval by the Ethics Committee of the Technische Universität Dresden. Following written informed consent, genomic DNA from the index patient was enriched with a SureSelect Human All Exon Kit V6 (Agilent technologies, Santa Clara, California) for subsequent exome sequencing on a HiSeq2500 (Illumina, San Diego, California). Reads were aligned to the GRCh37/hg19 build of the human reference genome. Variants were filtered by minor allele frequency, mode of inheritance and predicted functional impact using the VarFish platform [1]. For further prioritization of variants, we used the following HPO terms [2]: muscle weakness—HP:0001324, dysarthria—HP:0001260, hyperreflexia—HP:0001347, elevated serum CK—HP:0003236, fasciculations—HP:0002380 and skeletal muscle atrophy—HP:0003202. Detected variants were classified according to the guidelines of the American College of Medical Genetics [3]. Co-segregation was performed using Sanger Sequencing. Variant analysis identified two pathogenic variants in *HEXA* (NM_000520.6): c.508C>T, p.(Arg170Trp) and c.805G>A, p.(Gly269Ser) in compound heterozygous state, leading to the rare diagnosis of late-onset Tay-Sachs disease (LOTS; Fig. 1).

Fluorometric measurements of the lysosome isoenzyme HexA and HexB activity in leucocytes using 4-methylumbelliferyl derivatives confirmed a severely reduced HexA activity of 2% (0.03 nmol/l/min, reference 0.96–1.78), while HexB activity was within normal range, consistent with LOTS.

Prior to establishment of the patient's diagnosis, a neuropsychological assessment at the age of 16/17 years, using subtests of the German adaptations of either the Wechsler Adult Intelligence Scale-Revised (HAWIE-R) and the Cattell’s Culture Fair Test (CFT20-R) assigned his processing speed, verbal working memory performance, perceptual reasoning, semantic knowledge and general intelligence to the lower limit of the age-adjusted norm.

Tay-Sachs disease (TSD, MIM #272800) is a rare autosomal recessive progressive neurodegenerative disorder caused by pathogenic variants in *HEXA* (hexosaminidase A gene, MIM *606869) that impairs β-hexosaminidase A (HexA) enzyme activity, resulting in lysosomal accumulation of GM2 gangliosides within the central nervous system. The most common severe infantile form leads to early childhood death, owing to lack or negligible enzyme activity [4]. Late-onset forms of TSD result of varying grades of preserved residual enzyme activity manifesting with a chronic clinical course of disease. Progressive degeneration of upper and lower motor neurons with onset before 25 years of age may mimic juvenile amyotrophic lateral sclerosis (JALS) [5].

While the specific combination of the patient’s pathogenic variants in *HEXA* has to our knowledge not yet been described, the first variant c.508C>T was reported in association with the infantile course of TSD with a complete loss-of-function in *in-vitro* assays [6]. A detailed characterization of the second variant c.805G>A, has recently been described by Jahnova et al. to provoke later-onset forms of disease owing to a residual HexA activity of 1.8–4.1% [7]. In a systemic Orphanet-survey performed in 2020 the overall birth prevalence of TSD was estimated at 0.28/100,000 [8]. Considering the limited number of patients carrying the c.805G>A variant in *HEXA* previously published, the presence of this specific variant within a cohort of 14 Czech LOTS patients is astonishing [7]. While a geographical clustering of the variant c.805G>A cannot be ruled out, the numbers suggest that TSD and especially its late-onset form (LOTS) still remain widely under- or misdiagnosed. In the case described here, JALS was initially suspected, because the clinical subacute progression of upper and lower motor neuron compromise fulfilled the revised El Escorial criteria for probable ALS [5]. A comparative analysis results in one indicator that enables a distinction between disease JALS and LOTS, i.e. atrophy of the cerebellum (Table 1). Notably, all 14/14 LOTS patients described by Jahnova et al*.* as well as all 18/18 LOTS patients reported by Neudorfer et al*.* exhibit the clinical finding of cerebellar atrophy [4, 7].

**Table 1:** Differential diagnosis of late-onset Tay-Sachs and juvenile amyotrophic lateral sclerosis compared with the symptoms presented by the index patient

Symptom	LOTS	JALS	Index patient
Age of onset (years)	10–36	11–19	13
Cachexia	−/ +	+	+
Dysarthria	+	+	+
Muscle weakness	+	+	+
Muscle atrophy	+	+	+
Muscle fasciculations	+	+	+
Hyperreflexia	+	+	+
Cerebellar ataxia	+	+	+
Cerebellar atrophy	+	−	+
Tremor	+	−	+
Cognitive impairment	+	−	+
Elevated serum CK	(+)	−	+

*LOTS* late-onset Tay-Sachs, *JALS* juvenile amyotrophic lateral sclerosis

LOTS patients may frequently manifest with debilitating neuropsychiatric features and/or cognitive dysfunction. Another recent study revealed that 7/7 LOTS patients, all of whom also exhibited considerable global cerebellar atrophy on MRI, showed evidence of cerebellar cognitive affective syndrome (CCAS) [9]. CCAS has been attributed to affection of the cerebellar posterior lobe and may occur separately or together with cerebellar motor- and vestibular syndrome. As cognitive cerebellar syndrome, it is believed to reflect dysmetria of thought, analogous to dysmetria of motor control, impairing executive, linguistic and visuospatial functions as well as regulation of emotion and affect [10]. CCAS was identified using a newly developed and validated CCAS syndrome scale, specifically designed for the expedited clinical assessment of cognition and neuropsychiatric features in patients with cerebellar disorders [11].

Accurate and early diagnosis is becoming increasingly relevant with new treatment options emerging. Investigations of various therapeutic strategies have shown encouraging results and motivated their advancement to clinical trials. Currently active interventional studies listed in the ClinicalTrials.gov database include hematopoietic stem cell transplantation (Enriched hematopoetic stem cell infusion: phase 1/2, USA, NCT01372228), enzyme replacement therapy, substrate reduction therapy (Miglustat: phase 3, Iran, NCT03822013; Venglustat: phase 3, USA, NCT04221451; Miglustat and ketogenic diet: phase 4, USA, NCT02030015), umbilical cord blood (UBC) transplantation (UBC-derived oligodendrocyte-like cells, phase 1, USA, NCT02254863), cerebellar ataxia treatment (N-Acetyl-L-Leucine: phase 2, USA, NCT03759665) and gene therapy (AXO-AAV-GM2: phase 1, USA, NCT04669535).

Since diagnosis identification is the first, yet decisive prerequisite for the advancement of an effective therapy, emphasis on the clinical findings of atrophy and compromise of the cerebellum in LOTS diagnostic guidelines may facilitate diagnosis.
